# Supplementary material for: Genome-wide identification, classification and expression profile analysis of the HSF gene family in Hypericum perforatum
Source: PeerJ. 2021 May 6;9:e11345. doi: 10.7717/peerj.11345 (PMC8106910; doi:10.7717/peerj.11345)
Supplement: Supplemental Information 2 [file peerj-09-11345-s002.docx]

**Table S2. List of primers used for qRT-PCR analysis.**

|  | **Forward primer** | **Reverse primer** |
| --- | --- | --- |
| *HpHSF01* | TCCAGAGGCGGAAGGTGTCA | GTTGCTGGAGACCGCCAGTT |
| *HpHSF02* | TGAGCTCCACGCCATCGAGA | CCCAGAAGCCATCCTCCAGGT |
| *HpHSF03* | CCAGCGCCGTTCCTAACCAA | GGAAGAAGGTCCCTCGCGAAC |
| *HpHSF04* | CGAGGTCGTCTCGTGGAACG | GCTTGCGAAGTTGCTGTGCT |
| *HpHSF05* | TGGCAACAAGCCGATGGGAA | TGTTCCGACGAGGTCGAGGT |
| *HpHSF06* | GGCAACAAGCCGATGGGAGT | TGTTCCGACGAGGTCGAGGT |
| *HpHSF07* | GCGCCGTTCTTGCTGAAGAC | ACTCCGGAGGGTTCCAAACGA |
| *HpHSF08* | ACGGCTTCGTCGTCTTCGTC | GCCTCGAGTCGCTTGCTCAT |
| *HpHSF09* | GCAAGGCCGACAACAGCTTC | ACCACTCGCTCGCAAACTCC |
| *HpHSF10* | GGCTGCTGGGCCTTCTCTTG | CACGTTGCTGCTGTCGCAAT |
| *HpHSF11* | TGCCCATTTCGGGACGAAGC | CGGGATCTCGCGGAAGCATT |
| *HpHSF12* | TGCTAGCTCCCTACCGCCTT | GCAAACTCGGGTGGGTTCCA |
| *HpHSF13* | GGCTGAGCCAGGAAGCATCA | GCGACTGTTTGCAGCACTGG |
| *HpHSF14* | GTGGTCACCGGCCGATTTCT | CCAGCATGTGCCTGCTTCCT |
| *HpHSF15* | CATCGCAGCTGGTGGAGGAG | CCCGACCTTGCAATGCCTCA |
| *HpHSF16* | CAGCTTCGTCCGCCAGCTTA | GAGCAGGCTTTCTCCGGCTT |
| *HpHSF17* | GCTGCAGCATGCATCGAAGTG | TGCCGCTGCTCCATCATCTG |
| *HpHSF18* | GTCTCAATAGCCCGGCTGCAT | CCCAGTGGCCACAGTTGGAG |
| *HpHSF19* | AGCCCAGCTCCTTTCTTGGC | AGCTCGGAGAAATCAGCCGTTG |
| *HpHSF20* | ATCTCAGCCGGTTGGGTTGC | CCGGACCGTTCCATGTTGCT |
| *HpHSF21* | TGGGTGCGAGTGCATGTGTT | GCTGCCGTTGCTCCATTCCT |
| *HpHSF22* | GGCGCCGTTCCTAACCAAGA | GGAAGAAGGTCCCTCGCGAAC |
| *HpHSF23* | TCACCATGTGGCTGCTGCTT | TGTGGTCTGTGGTTGGGTCCT |
